# *Euonymus chengduanus* (Celastraceae), a New Species Unexpectedly Discovered in an Urban Forest Park in the Megacity of Chengdu, West China

**DOI:** 10.1002/ece3.71234

**Published:** 2025-04-10

**Authors:** Jun Hu, Xin Huang, Yao Luo, Qiurong Liu, Hai He, Qing Liu

**Affiliations:** ^1^ Ecological Restoration and Biodiversity Conservation Key Laboratory of Sichuan Province Chengdu Institute of Biology, Chinese Academy of Sciences Chengdu China; ^2^ University of Chinese Academy of Sciences Beijing China; ^3^ College of Life Sciences, Chongqing Normal University Chongqing China

**Keywords:** Celastraceae, degraded ecosystem, *Euonymus*, *Glyptopetalum*, new species, urban

## Abstract

The establishment of native species‐dominated forest parks and investment into vegetation surveys and taxonomic research has not only enabled the discovery of relictnative species affected by urban expansion but also facilitated targeted conservation efforts. During investigations on plant diversity and vegetation status in Longquan Mountain Urban Forest Park in eastern Chengdu Metropolis, West China, an unidentified species of *Euonymus* (Celastraceae) was encountered. It resembles both *E. chloranthoides* and *E. aquifolium* in vegetative appearance, but it has exclusively 4‐merous flowers. A phylogenetic analysis incorporating 51 taxa of *Euonymus* and *Glyptopetalum* supported its recognition as a new species, positioned phylogenetically sister to a clade containing *Glyptopetalum* species, and close to *E. chloranthoides* and *E. aquifolium*. To acknowledge the expanded Chengdu urbanization and the recently planned mountain urban forest park, it is described as *E. chengduanus*, and its diagnostic morphological characters are compared with *E. aquifolium*, *E. chloranthoides*, and *Glyptopetalum ilicifolium*. This discovery implies that even in intensively anthropogenic‐impacted areas adjacent to large cities, undocumented plant diversity may still exist. Consequently, this new species is tentatively assessed as Critically Endangered (CR) to emphasize both the urgency of its protection and to raise public awareness concerning biodiversity in degraded ecosystems.

## Graphical abstract

This study discovered a new plant species within the urban area and named it Chengdu for the first time. This discovery implies that even in intensively anthropogenic‐impacted areas adjacent to large cities, undocumented plant diversity may still exist.

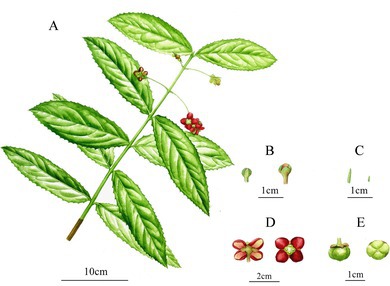

## 1 Introduction

The Longquan Mountains, located in the western part of the Sichuan Basin and on the eastern fringes of the Chengdu flatland in Southwest China, span approximately 200 km from northeast to southwest and are typically 10 km wide. They serve as the divide between the Minjiang and Tuojiang Rivers, two of the largest tributaries of the Yangtze River in Sichuan Province. Despite the Longquan Mountains reaching a peak elevation of 1051 m, the majority of their area lies within an elevation range of 400–800 m. Historically, the native northern subtropical evergreen forests in these regions have been largely replaced by croplands, orchards, and diverse artificial forest stands or bamboo groves (Sichuan Vegetation Cooperation Group 1980).

Over the years, Chengdu has developed into a metropolis, and its urban expansion has reached the Longquan Mountains. In 2017, the central section of the Longquan Mountains, about 90 km in length and just east of the expanded urban area of Chengdu, was designated as the Longquan Mountain Urban Forest Park. Since then, a multitude of surveys and investigations have been conducted in this area to acquire fundamental data on biodiversity and urban ecology for the construction of this forest park.

Based on recent comprehensive plant species collections and related vegetation surveys in the Longquan Mountain Urban Forest Park, a total of 628 vascular plant species have been documented (Hou et al. 2021; Yu et al. 2023). Although this represents an incomplete account of the plant species in the area, the number is considerably smaller compared with that of Jinyun Mountain in Chongqing. Jinyun Mountain, with lower summits and a smaller area, still harbors around 1700 vascular species (Xiong 2005). New individual distributional records of certain taxa have been noted (e.g., Luo et al. 2022), and the probability of discovering new species in this fragmented ecosystem has been low. However, during one of our surveys in 2021, an unknown plant of *Euonymus* Linnaeus (1753, 197) was discovered on a cliff within a vegetation area severely affected by human activities. It differs from the three wild species commonly encountered in the same area: *E. acanthocarpus* Franchet (1889, 129), *E. fortunei* (Turczaninow 1863, 603; Handel‐Mazzetti 1933, 660), and *E. grandiflorus* Wall. in Roxburgh (1824, 404). When comparing its vegetative and fruiting morphology with that of Celastraceae species in Sichuan and neighboring regions that have similar spiny, leathery leaves, such as *E. aquifolium* Loes. & Rehder in Sargent (1911–1913, 484), *E. chloranthoides* Yang (1945, 90), and *Glyptopetalum ilicifolium* (Franchet 1886, 453; Cheng 1999, 92; Chang 1988; Cheng 1999; Ma et al. 2008; Hu, Zhang, et al. 2022), its identity remained uncertain.

Fortunately, in May 2022, flowering specimens were collected during a revisit to the site. Its 4‐erous flowers and the color of the floral disc clearly suggested it was a new species. To determine whether the differences in its floral and fruit characteristics are sufficient to justify the description of a new species, molecular data were generated for this potential new species and for *E. chloranthoides* from its type locality. It is also hypothesized that this new species will be grouped into a clade with species documented in *Glyptopetalum* Thwaites (1856, 267) in Chinese floras.

## 2 Materials and Methods

### 2.1 Morphological Observations

Morphological information of this new species was obtained from observations and measurements of living plants in the field, and dried herbarium specimens were deposited at CDBI (hereafter acronym of herbarium follows Thiers 2024). Physical specimens of *Euonymus* at CDBI were thoroughly examined, and virtual specimens of related species available at other herbaria (i.e., A, BNU, CSH, HGAS, IBK, IBSC, IMC, KUN, P, PE, and SM) were also searched. The terminology of Harris and Harris (2001) was consulted for general morphology, the definition of Hickey (1973) and Ellis et al. (2009) was referred to for leaf venation, and the characteristic order of Ma et al. (2008) was followed for species description.

### 2.2 DNA Extraction and Sequencing

The sequences of this new species and *Euonymus chloranthoides*, a morphologically closely related species (vouchers: *Jun‐Yi Zhang ZJY233*, CDBI), were newly obtained in the procedure involving the following steps. Total DNA was extracted from silica‐gel dried leaves via a Plant DNA Isolation Kit (Cat. No. DE‐06111, Foregene, Chengdu, China). The sequences of nuclear internal transcribed spacer (nrITS) were amplified using the same primers as previous phylogenetic studies on *Euonymus* (referring to Li et al. 2014; Hu, Zhang, et al. 2022). The PCR program consisted of an initial 4 min preheating stage at 96°C, followed by 35 cycles of 30 s at 96°C (denaturation), 30 s at 50°C–58°C (annealing) and 60–100 s at 72°C (extension), and lastly by a final 8 min extension at 72°C. The PCR products were sent to TSINGKE Biotech (Chengdu, China) for sequencing. The returned sequences were edited via Sequencher v4.1.4 (Gene Codes, Ann Arbor, Michigan, USA) and checked manually, and then deposited in GenBank with the accession numbers OR725028 and OR725029, respectively for this new species and *E. chloranthoides*.

### 2.3 Phylogenetic Analyses

The phylogenetic analysis included 58 accessions, which represented 55 taxa including two species of *Celastrus* Linnaeus (1753, 196) and two identities of *Tripterygium* Hook.f. in Bentham & Hooker (1862, 360) as outgroups. Seven species in the 51 entities included in the ingroup were documented as *Glyptopetalum* Thwaites (1856, 267) in Ma et al. (2008) following the previous study of Hu, Zhang, et al. (2022). Information on taxa and accessions in GenBank was summarized in Appendix S1. All sequences were aligned using MAFFT v7.475 (Katoh and Standley 2013) with default parameters. Bayesian inference (BI) and Maximum likelihood (ML) analyses were performed to infer the phylogenetic relationships within combined datasets. For BI analyses, the nucleotide substitution models for these data matrices were estimated using the software jModeltest v2.1.6 (Posada 2008) and the best fit models (GTR + G) were selected using the corrected Akaike Information Criterion (AICc). The BI analysis was conducted using MrBayes v3.2.7a (Ronquist and Huelsenbeck 2003), with two separate Markov‐chain Monte Carlo (MCMC) chains (1000,000 generations and sampled every 1000 generations). The first 25% of the trees were discarded as burn‐in, and the remaining trees were used to generate a majority‐rule consensus tree. We performed ML analyses using IQ‐TREE v1.4.2 (Nguyen et al. 2014) with branch support estimated using 2000 replicates of both SH‐like approximate likelihood‐ratio test (SH‐aLRT) (Guindon et al. 2010) and the ultrafast bootstrapping algorithm (UFboot) (Minh et al. 2013). The resulting phylogenetic trees were visualized using FigTree v.1.4.4 (https://github.com/rambaut/figtree/releases/tag/v1.4.4).

## 3 Results

**FIGURE 1:**
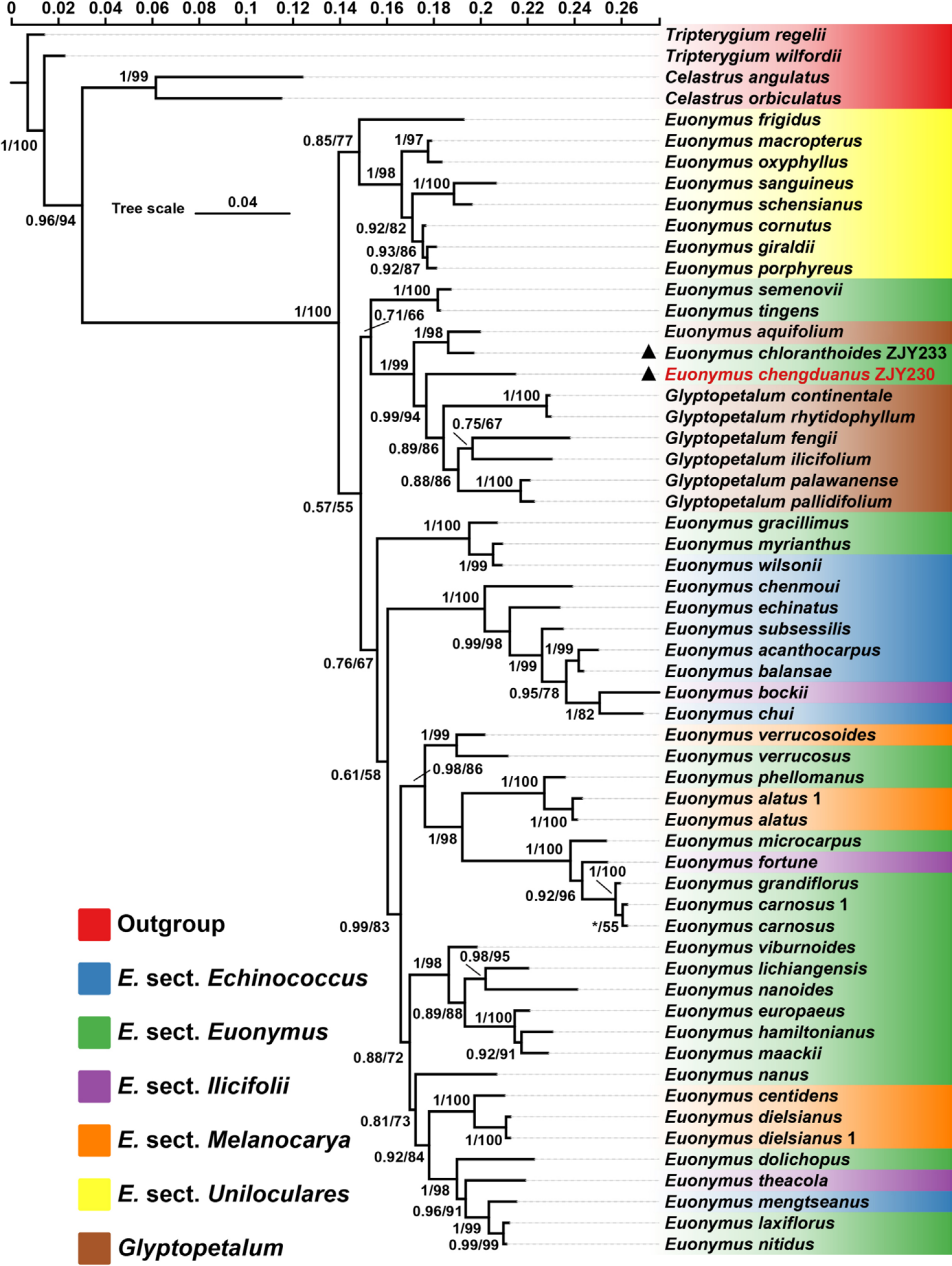
Bayesian and ML phylogenetic tree based on nuclear internal transcribed spacer (ITS) of the 58 accessions representing 55 taxa of *Euonymus* and/or *Glyptopetalum*. Values above branches are Bayesian posterior probabilities (> 0.5)/maximum likelihood bootstrap percentages (> 50). Terminal nodes are in seven different colors corresponding to the five sections of *Euonymus* and the genus *Glyptopetalum* described in *Flora of China* (Ma et al. 2008), as well as the outgroup. Solid delta marks the newly generated sequence in this study.

The topography of the molecular phylogenetic tree (Figure 1) showed overall similarity with previous studies (e.g., Simmons et al. 2012; Li et al. 2014; Hu, Zhang, et al. 2022), which revealed the monophyly of *Euonymus* in a broad sense. The infrageneric division remained inconclusive in that of the five sections adopted in the Chinese floras (Cheng 1999; Ma et al. 2008), only species in *E*. sect. *Uniloculares* Rouy and Foucaud (1893–1913, 159) were clustered together with moderate support (BI/ML = 0.85/77). The newly found species was sister to a well‐supported subclade (BI/ML = 0.99/94), containing six *Glyptopetalum* species (BI/ML = 1/99, Figure 1), which further clustered with a clade consisting of *E. chloranthoides* and *E. aquifolium*. Interestingly, the clustering of *E. chloranthoides* with *E. aquifolium* corroborates Yang's (1945) assumption of their morphological similarity when describing the former taxon.

**FIGURE 2:**
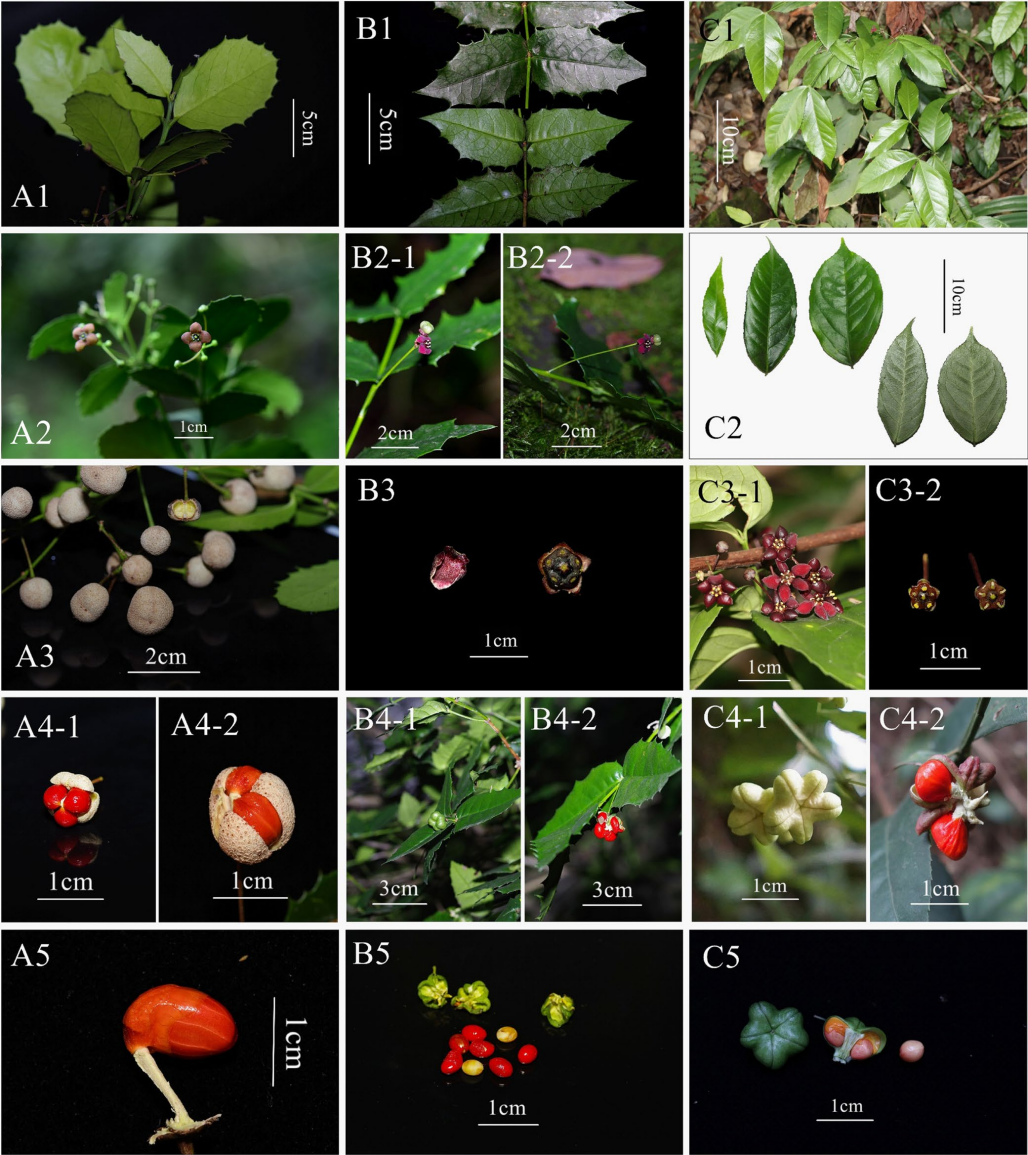
Three closely related species of *Euonymus chengduanus* (A) *Glyptopetalum ilicifolium* (A1) Branch, (A2) flower, (A3) capsule, (A4) cracked capsules, (A5) seed with aril; (B) *Euonymus aquifoliuum* (B1) Leaves, (B2) part of floral branch showing a flower, (B3) a detached petal (the left) and disk and calyx (the right), (B4) fruits at different developmental stages, (B5) mature seeds; (C) *Euonymus chloranthoides* (C1) Branch, (C2) leaves, (C3‐1) flowers, (C3‐2) flower discs at different developmental stages, (C4) uncracked and cracked capsules, (C5) fruits and seeds. (A2, C1), (A5), and (C4, C5) were photographed by Hong Jiang, Junyi Zhang, and Feng Chen, respectively. (B1–B3) were from Hu, Zhang, et al. (2022). The other photos were photographed by Jun Hu.

Based on the phylogenetic inference and morphological comparison with three related species distributed in Sichuan, Chongqing, Guizhou, and Yunnan: *Euonymus aquifolium*, *E. chloranthoides*, and *Glyptopetalum ilicifolium* (Table 1 and Figure 2, and referring to selected additional specimens examined in the following taxonomic treatment), and the previous discussed rationale of generic placement for *Euonymus aquifolium* (Hu, Zhang, et al. 2022), this newly found plant is hereafter described in the genus *Euonymus*.

**TABLE 1:** Comparisons among *Euonymus aquifolium*, *E. chengduanus*, *E. chloranthoides*, and *Glyptopetalum ilicifolium* (≡*E. ilicifolius*) in terms of morphology, habitat, and distribution.

Species	*E. aquiflium*	*E. chengduanus*	*E. chloranthoides*	*G. ilicifolium*
Plant height	0.6–1.8 m	0.6–1.5 m	1.2–2.5 m	1.5–2.8 m
Leaf texture	Hardened leathery	Thinly leathery	Hardened leathery	Thickly leathery
Leaf shape	Oblongly ovate, ovate to orbicularly ovate, base slightly cordate, apex acute to shortly acuminate	Narrowly ovate, ovately elliptic, or ovately lanceolate, base cuneate, apex acute to shortly acuminate	Obovate, oblong‐obovate, or elliptic to oblong‐elliptic, base acute to cuneate, apex acuminate	Obovate, elliptic, or narrowly elliptic, base broadly cuneate, apex rounded or obtuse
Leaf margins	Obviously uneven, with 6–11 spiny tipped coarse teeth each	Slightly uneven, with to 20 regularly spaced shortly spiny tipped teeth each	Almost even, with to 20 regularly spaced sharply tipped teeth each	Almost even, with 6–10 spiny tipped teeth each
Leaf venation	Semi‐craspedodromous, lateral veins 6–10 pairs	Festooned semicraspedodromous, lateral vein 4–7 pairs	Semi‐craspedodromous, lateral veins 7–9 pairs	Nealy craspedodromous, lateral veins 5–7 pairs
Petiole	Subsessile to 2 mm long	2–3 mm long	Subsessile or 1–2 mm long	2–6 mm long
Flora merosity	5‐merous	4‐merous	5‐merous	4‐merous
Sepal	Triangular, usually red‐brown throughout	Deltoid ovate, abaxially yellowish green	Broadly obovate, usually deeply red throughout	Very small, dark brown
Petal	Red‐brown to deep read, apex acute to sharply acute	Deep red or darkly read, apex obtusely acute	Red‐pink to black purple, apex acute	Dark brown, apex obtuse
Disk	Pentagonal, deeply red	Roundly quadrate, yellowish green except purply tinged lobes around ovary	Pentagonal, deeply red except lighter color around ovary	Quadrate, dark brown
Ovary	5‐locular with 2 ovules per locule	4‐locular with 2 ovules per locule	5‐locular with 2 ovules per locule	4‐locular with 1 ovule per locule
Fruit	Oblate globose; yellowish green when immature, glabrous	Nearly globose, yellowish green when immature, glabrous	Depressed globose in outline but usually 5‐lobed, yellowish green when immature, glabrous	Subgobose, pale yellowish brown, rather densely squarrose
Habitat	On or under cliff in deep valley, broadleaf or mixed forest, elev. 700–2200 m	Alongside cliff under secondary forest of degraded ecosystem, elev. ca. 800 m	Alongside cliff, stony slope under broadleaf forest, elev. mostly 200–500 m, rarely to ca. 1000 m	Dense forests, mountain slopes, elev. 500–2000 m
Distribution	West Sichuan	Eastern Central Sichuan	Chongqing (may extending to Adjacent eastern Central Sichuan)	Southwest Sichuan, Northwest Yunan, and West Guizhou

## 4 Taxonomy

**FIGURE 3:**
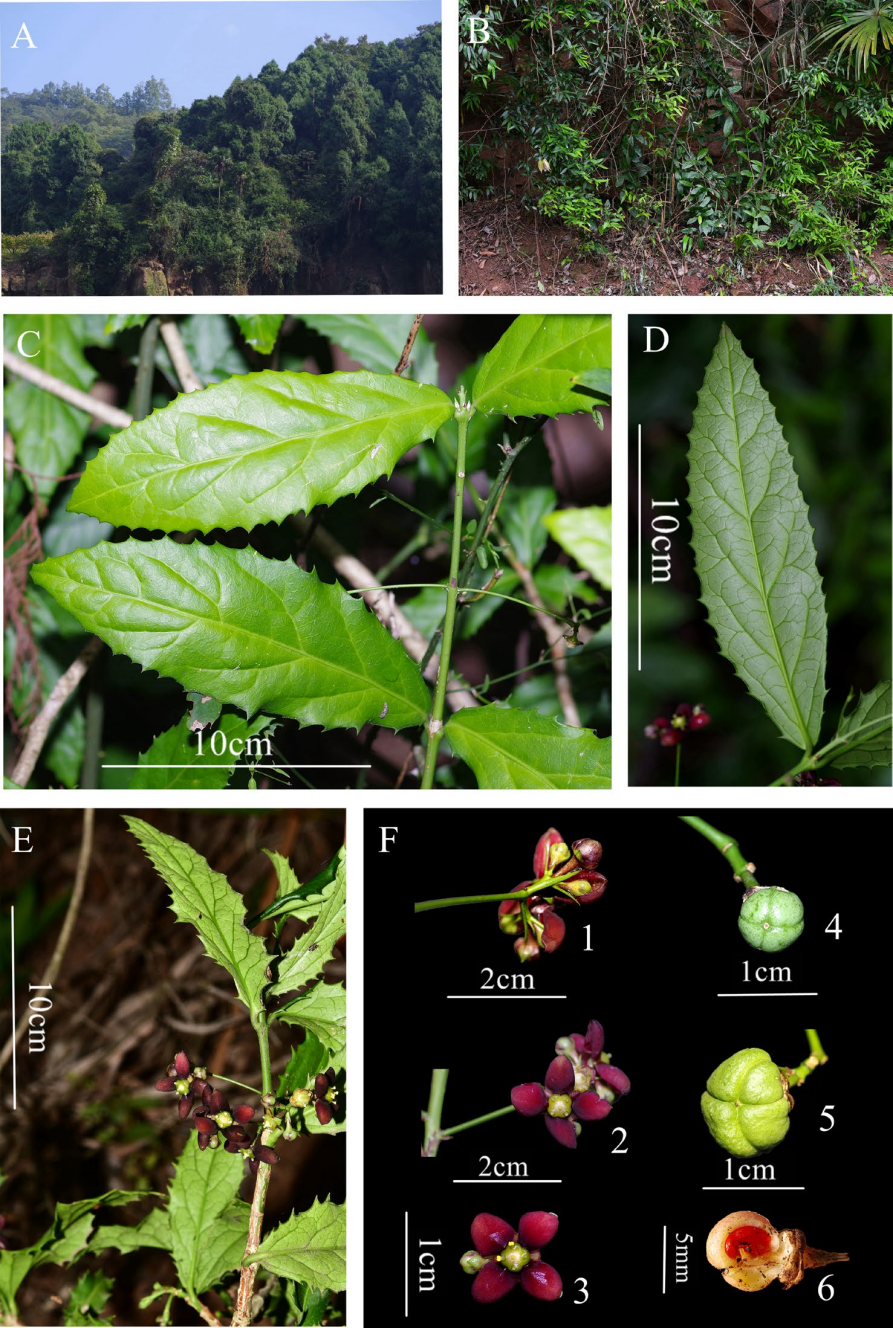
*Euonymus chengduanus*: (A) Habitat, (B) habit on cliff, (C) leaves in adaxial and abaxial view, (D, E) branch with flowers, (F) flowers and fruits; (F1) rear view of inflorescence, (F2) front view of inflorescence, (F3) a single flower, (F4–F6) fruits at different stages of maturity. (A–D, F) were photographed by Jun Hu; (E) was photographed by Yao Luo.

**FIGURE 4:**
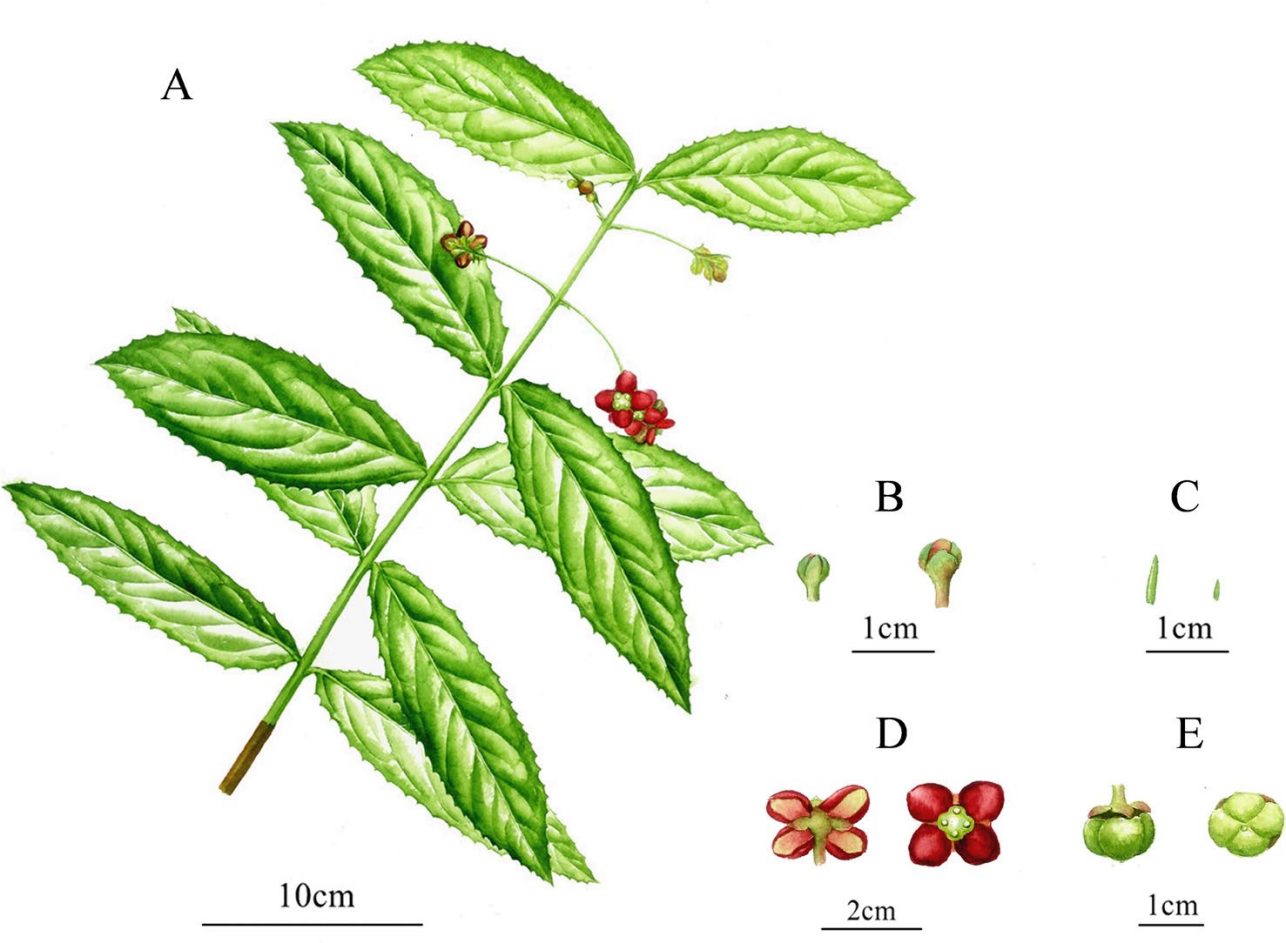
*Euonymus chengduanus* drawn by Congying Li based on specimens of *hujun20220527B01* (CDBI): (A) Branch, (B) flower buds from different periods, (C) bracteoles (the left) and bract (the right), (D) flower, (E) capsule.

*Euonymus chengduanus* J. Hu & H. He, sp. *nov*. (Figures 3 and 4).

### 4.1 Type

CHINA. Sichuan: Jianyang city, Jiajia residential district, Caiyuan Village, Longquan Shan (Longquan Mountain), N 30.5021°, E104.2981°, elev. ca. 808 m, 27 May 2022, J. Hu et al. *hujun20220527B01* (holotype CDBI (CDBI0298328!); isotype CDBI (CDBI0298327!)).

### 4.2 Diagnosis

This new species appears closely related to *Euonymus chloranthoides* in vegetative organs, inflorescence, and adjacent distribution, but it can be easily differentiated from the latter by its 4‐merous flowers, mostly light green floral disc, unlobed fruits, and a secondary vein framework showing festooned semicraspedodromous patterns. It also shares a certain extent of similarity to both *E. aquifolium* and *Glyptopetalum ilicifolium*, and these four species can be distinguished from the morphological characters of leaf, flower, and fruit (Table 1 and Figure 2).

### 4.3 Description

Evergreen shrubs, 0.8–2 m tall, glabrous throughout; branchlets firstly 4‐angled from emergence, turning terete with hardening, color from green in the first 1–2 years to gray brownish or brown with age. Leaves opposite; petioles 2–3 mm long; leaf blades thinly leathery, slightly uneven on margins, narrowly ovate, ovate elliptic, or ovate lanceolate, 4–12 × 1.5–4.5 cm, base cuneate, margin with more or less regularly distributed spine‐tipped teeth, apex acute to shortly acuminate, adaxially shiny green, abaxially yellowish green; venation of festooned semicraspedodromous, lateral vein 4–7 pairs, primary and secondary veins obviously elevated abaxially, and networks of tertiary and quaternary veins visible on both surfaces. Cymes extra‐axillary, or sometimes axillary, opposite, 3–7 flowered; bract 1, lightly brown, subulate, 1–2 mm long, caducous; peduncle 1.5–4.5 cm long; pedicels 3–12 mm long, usually with a pair of opposite bracteoles; bracteoles dull green, lanceolate‐shaped subulate, 4–10 mm long, ca. 1 mm wide, mostly shedding in fruiting. Flower deep red or dark red, 1–1.5 cm in diameter, 4‐merous; sepals deltoid ovate, 3–5 × 4–6 mm, margin entire, apex sharply acute, abaxially yellowish green, or sometimes purplish tinged, slightly fleshy in texture, persistent; petals fleshy, ovate to broadly ovate, 5–9 × 4–6 mm, margin entire, apex obtuse and marginally revolute; disc quadrate with rounded angles and concave sides, yellowish green, centripetally thinner and roundly 4‐lobed, more or less purplish tinged around the surface of ovary; staminal cushion same color as the outer disc, filament very short, anther yellowish; upper exposed ovary pale yellow; style inconspicuous; stigma small, rounded; ovary 4‐locular; ovules 2 per locule. Capsules nearly globose, with shallow grooves along the surface of locular septa, 6–12 mm in diameter, 6–10 mm in height, yellowish green when immature. Mature seeds unseen, immature seeds covered with red aril over half their surface when fresh.

### 4.4 Phenology

Flowering in May (and might be from April to June); fruiting from post‐anthesis to September (immature).

### 4.5 Habitat and Ecology

*Euonymus chengduanus* was found alongside a small cliff, which is surrounded by small patches of cultivated *Cupressus funebris* Endl. forest, peach gardens, and groves of *Bambusa emeiensis* L. C. Chia & H. L. Fung. The accompanying tree and shrub plants include *Broussonetia papyrifera* (L.) L'Hér. ex Vent., *Debregeasia orientalis* C. J. Chen, *Lindera communis* Hemsl., *Myrsine semiserrata* Wall., and *Trachycarpus fortunei* (Hook.) H. Wendl. The intertwisted vines and climbers are *Asparagus lycopodineus* (Baker) F. T. Wang & T. Tang, *Cayratia japonica* (Thunb.) Gagnep., *Clematis chinensis* Osbeck, *Hedera nepalensis* var. *sinensis* (Tobler) Rehder, *Periploca forrestii* Schltr., and *Smilax microphylla* C. H. Wright. The understory herbs consist of, among others, *Achyranthes bidentata* Blume, *Aster falcifolius* Hand‐Mazz., *Boehmeria clidemioides* var. *diffusa* (Wedd.) Hand‐Mazz., *Crassocephalum crepidioides* (Benth.) S. Moore, *Equisetum ramosissimum* subsp. *debile* (Roxb. ex Vaucher) Hauke, *Iris japonica* Thunb., and *Setaria palmifolia* (J. König) Stapf. It is obvious that these coexisting species are mostly members of secondary succession or exotic and invasive species (referring to Deng et al. 2020), which indicates the intensive anthropogenic interferences in the local environment.

### 4.6 Etymology

The species epithet refers to the megapolis of Chengdu, and the Latin adjective ending‐*anus* indicating location. Chengdu is one the largest urban areas in western China with Thousands of years of history. Mountainous regions on the West of Chengdu hold huge biodiversity, where E. H. Wilson (referring to Sargent 1911–1913) had collected quite a lot living plants and specimens, and Wilson (1929) himself accredited Chengdu as “the Garden of West China”. However, no plant has been credited to the city name of Chengdu. Urbanization in recent decades has enlarged the city of Chengdu around all the way outside, and extending eastwards across Longquan Mountain to Jianyang City. This is the first plant species named after Chengdu, and a Chinese name “成都卫矛(Chéng dū wèi máo)” is here suggested.

### 4.7 Conservation Status

**FIGURE 5:**
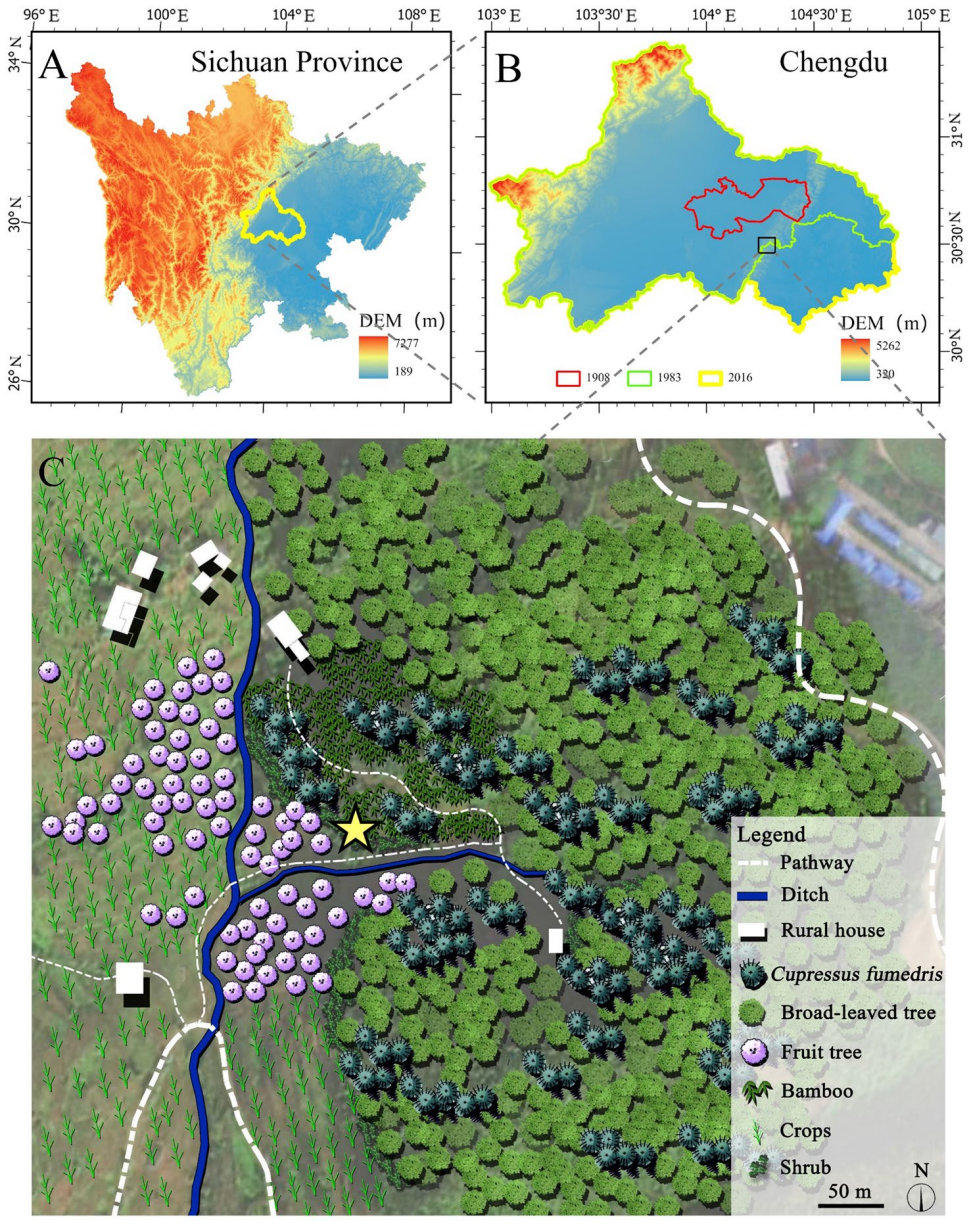
Distribution map of *Euonymus chengduanus* drawn by Fei Gao and Qinqin Guan (A). The scope of Sichuan Province in western China (B). The scope represented or governed by the big city of Chengdu in different historical periods (C). The situation of degraded ecosystems around the new species. The position of the yellow pentagram indicates the distribution location of *Euonymus chengduanus*.

*Euonymus chengduanus* is presently only known from the type locality, where ca. 10 individuals occupy an area of ca. 25 m^2^ (Figure 5). It was not found in similar habitats by recent extensive vegetation investigations and other related expeditions for planning and implementing the Longquan Mountain Urban Forest Park (Hou et al. 2021; Yu et al. 2023). This new plant is temporarily assessed as Critically Endangered (CR) based on the IUCN Standards and Petitions Committee (2022) categories and criteria to highlight its conservation emergency. It is hopefully expected that the endemicity of this new species will promote public awareness concerning biodiversity protection even in degraded ecosystems.

### 4.8 Selected Additional Specimens Examined

#### 4.8.1 Euonymus Chengduanus

CHINA. Sichuan: Jianyang city, Jiajia residential district, Caiyuan Village, Longquan Shan (Longquan Mountain), N 30.5021°, E104.2981°, elev. ca. 808 m, 18 September 2021, Y. Luo et al. *luoyao20210918B01* (paratype CDBI (CDBI0298325! & CDBI0298326!)).

#### 4.8.2 Euonymus Aquifolium

CHINA. Sichuan: Jinkouhe District [Wa‐shan], on cliffs, elev. ca. 2200 m, November 1908, *E. H. Wilson 1366* (holotype A00049691; isotypes K000669647 & US00096036); Jiulong County, Wanba village, Hei Dong Zi Gou, elev. ca. 1850 m, 10 August 2021, *hujun20210810B01* (CDBI!).

#### 4.8.3 Euonymus Chloranthoides

CHINA. Chongqing: Beibei [Peipei], Wenquan [Won‐juan], Jinyunshan, 21 February 1938, *K. L. Chu 6658* (holotype PE 00022736; isotopes IBK), July 1939, *L. Y. Lin 1905* (PE 00797116), September 1940, *L. Y. Lin s.n*. (PE 00022737), [Pa Hsian], 18 March 1934, T. T. Yu 2820 (PE 00797117); Nanchuan, Jinfoshan, elev. ca. 750 m, 15 August 1986, *Jinfoshan Exped. 2752* (PE 00797112), elev. ca. 650 m, 12 November 1997, *Z. Y. Liu 30,081* (IMC0095262), elev. ca. 980 m, 21 October 2008, *Z. Y. Liu 31,845* (IMC0095263), elev. ca. 750, 21 October 2008, *Z. Y. Liu 12,886–01* (IMC0095332); Bishan, *Bishan Exped. 305* (SM709500688); Tongliang, *Tongliang Exped. 295* (SM709500682); Dazu, *Dazu Exped. 667* (SM709500681).

#### 4.8.4 Glyptopetalum Ilicifolium

CHINA. Yunnan: Heqing [Ta pin tze], Da Long Tan [Ta long tan], 3 March 1886 *Delavay 1932* (possibly holotype P‐P00270639); 1913, *G. Forrest 11,415* (PE 00827734), August 1914, *G. Forrest 13,085* (PE 00827732); 14 December 1940, *T. N. Liou 17,710* (IBSC 0286017), 25 December 1946, *T. N. Liou 22,254* (IBSC 0286016); Lijiang, 12 September 1939, *K. M. Feng 2554* (KUN 0414022); Yongsheng Xian, elev. ca. 1455 m, 30 June 2015, *J. C. Hao 15,014* (BNU 0026611). Guizhou: Shuicheng City, Yezhong Xiang, Mawo Zhai, elev. ca. 1250 m, 30 September 1987, *Q. H. Chen 229* (HGAS 026682). Sichuan: Butuo Xian, J. Cai et al. *21CS20304* (KUN 1551126); Xichang City, 2021, *C. B. Ma & D. L. Lin YDYC137* (CDBI).

## 5 Discussion

Although a series of evidence suggests that the genera *Glyptopetalum* and *Euonymus* form a monophyletic group (Simmons et al. 2012; Li et al. 2014; Hu, Zhang, et al. 2022), the specific relationship between these genera requires further research due to the limited sampling of *Glyptopetalum* species in this study and the lack of material for *Glyptopetalum zeylanicum* Thw., the type species of the genus. However, the discovery of *Euonymus chengduanus*, coupled with observations of mature fruit morphology in *Euonymus aquifolium* (Figure 2B4 and B5), revealed two distinct fruit types within the subclade containing *Glyptopetalum* species. One type is flattened spherical, yellow green, smooth and spot free, represented by *E. chengduanus*, *E. aquifolium*, and *E. chloranthoides*. The other type is round spherical, gray white, with a surface densely covered with small patches of chaff, represented by *G. ilicifolium*, *G. rhytidophyllum*, and *G. continentale*. These findings may provide new information and reference for resolving phylogenetic relationships within the genus *Euonymus* in future studies.

In comparison to other renowned mountains in Sichuan, such as Emei Mountain (elev. 3099 m) and Gongga Mountain (elev. 7556 m), Longquan Mountain has a lower elevation and relatively poor biodiversity. In the past, it was not a hotspot or key area for field investigations and plant diversity research. New species or records in Sichuan have typically been concentrated in the Hengduan Mountains or the southern Jinsha River valley area (Hu, Liu, et al. 2022; Zhang et al. 2022; Hu et al. 2023; Liang et al. 2024; Yu et al. 2024). However, recent survey data have revealed traces of remaining subtropical native vegetation in some valleys (Tang et al. 2020; Yu et al. 2023), and the discovery of *Euonymus chengduanus* further substantiates this. At present, Longquan Mountain is undergoing the establishment of an Urban Landscape Forest aimed at building a park city, and there are also plans to construct the Chinese National Botanical Garden (Huang et al. 2019; Wan et al. 2019; Huang and Liao 2022). Consequently, *Euonymus chengduanus* will require further on‐site protection and breeding research in this area in the future.

To the east of Longquan Mountain, connected to Chongqing, lies a cluster of cities, making it one of the four major urban agglomerations in China. These areas are mostly characterized by low mountains and hilly terrain, and due to the influence of agricultural cultivation, native vegetation is typically distributed in a patchy and fragmented manner across some local valleys, receiving less research attention. Notably, multiple endemic species are found in these hilly regions (*Machilus salicoides* S. Lee, *Diospyros sutchuensis* Yang, *Rhododendron nymphaeoides* W. K. Hu, etc.) (Anonymous 2023). Given that this area serves as a disturbance zone caused by human activities and an ecological barrier zone for the Chengdu‐Chongqing Twin‐City Economic Circle (Zhao et al. 2021; Han et al. 2023), we suggest conducting further in‐depth investigations and research on the biodiversity and vegetation ecological data of this region in future work, in order to provide more scientific guidance for the construction of ecological barriers in this area.

## Author Contributions

**Jun Hu:** formal analysis (equal), investigation (lead), writing – original draft (equal). **Xin Huang:** data curation (equal), formal analysis (supporting), writing – review and editing (equal). **Yao Luo:** investigation (equal). **Qiurong Liu:** formal analysis (supporting), investigation (supporting). **Hai He:** formal analysis (lead), writing – original draft (equal). **Qing Liu:** resources (lead), writing – review and editing (equal).

## Conflicts of Interest

The authors declare no conflicts of interest.

## Supporting information


Appendix S1.

## Data Availability

DNA sequences: GenBank accessions: OR725028 and OR725029.
