# Supplementary material for: Euonymus chengduanus (Celastraceae), a New Species Unexpectedly Discovered in an Urban Forest Park in the Megacity of Chengdu, West China
Source: Ecol Evol. 2025 Apr 10;15(4):e71234. doi: 10.1002/ece3.71234 (PMC11985356; doi:10.1002/ece3.71234)
Supplement: Supplementary file 1 — Appendix S1. [file ECE3-15-e71234-s001.docx]

**Supplementary data**

Appendix A. The GenBank accessions for DNA sequences used in this paper with newly generated sequences in bold face.

| Taxon, GenBank accession No. for ITS. |
| --- |
| *Euonymus acanthocarpus* Franch*.*, KF282154; *E. alatus* (Thunb.) Siebold, EU328755 (1), MH710860 (2); *E. aquifolium* Loes. & Rehder, OK172405; *E. balansae* Sprague, KF282157; *E. bockii* Loes., KF282158; *E. carnosus* Hemsl., HQ393712 (1), KF282160 (2); *E. centidens* H.Lév., KF282161; *E. chenmoui* W.C.Cheng, KF282162; ***E. chloranthoides* Yen C.Yang, OR725029;** *E. chui* Hand.-Mazz., KF282163; *E. cornutus* Hemsl., JQ424143; *E. dielsiana* Loes. ex Diels, KF282166 (1), KF282167 (2); *E. dolichopus* Merr. ex J.S.Ma, KF282168; *E. echinatus* Wall., KF282169; *E. europaeus* L., HQ393713; *E. fortunei* (Turcz.) Hand.-Mazz., KF282177, *E. frigidus* Wall., KF282171; *E. giraldii* Loes., KF282172; *E. gracillimus* Hemsl., KF282173; *E. grandiflorus* Wall., HQ393711; *E. hamiltonianus* Wall., HQ393708; *E. laxiflorus* Champ. ex Benth., KF282178; *E. lichiangensis* W.W.Smith, MH117524; *E. maackii* Rupr., KF282180; *E. macropterus* Rupr., KF282181; *E. mengtseanus* Sprague, KF282182; *E. microcarpus* (Oliv. ex Loes) Sprague, KF282183; *E. myrianthus* Hemsl., KF282184, KF282185, KF282186; *E. nanoides* Loes. ex Rehder, KF282187; *E. nanus* M.Bieb., HQ393709; *E. nitidus* Benth., KF282188; *E. oxyphyllus* Miq., KF282191; *E. phellomanus* Loes., KF282193; *E. porphyreus* Loes., KF282195; *E. sanguineus* Loes., KF282197; *E. schensianus* Maxim., MH710838; *E. semenovii* Regel & Herder, KF282199; *E. sp.*, KF282200; *E. subsessilis* Sprague, KF282201; *E. theacola* C.Y.Cheng ex T.L.Xu & Q.H.Chen, KF282203; *E.* *tingens* Wall., KF282204; *E.* *verrucosoides* Loes., KF282206; *E.* *verrucosus* Scop., KF282205; *E.* *viburnoides* Prain, KF282207; *E.* *wilsonii* Sprague, KF282208; ***E. sp. nov.* ZJY230, OR725028**; *Glyptopetalum continentale* (Chun & F.C.How) C.Y.Cheng & Q.S.Ma, KF282210; *G. fengii* (Chun & F.C.How) Ding Hou, MZ568391; *G. ilicifolium* (Franch.) C.Y.Cheng & Q.S.Ma, OM985812; *G.* *palawanense* Merr., HQ393705; *G.* *pallidifolium* (Hayata) Q.R.Liu & S.Y.Meng, KF282192; *G.* *rhytidophyllum* (Chun & F.C.How) C.Y.Cheng, KF282211; *Celastrus orbiculatus* Thunb., KF282209 (1), KF282153 (2); *Tripterygium regelii* Sprague & Takeda, KF282212; *T. wilfordii* Hook. f., KF282213. |
